# A Genome Wide Association Study of arabinoxylan content in 2-row spring barley grain

**DOI:** 10.1371/journal.pone.0182537

**Published:** 2017-08-03

**Authors:** Ali Saleh Hassan, Kelly Houston, Jelle Lahnstein, Neil Shirley, Julian G. Schwerdt, Michael J. Gidley, Robbie Waugh, Alan Little, Rachel A. Burton

**Affiliations:** 1 ARC Centre of Excellence in Plant Cell Walls, School of Agriculture, Food and Wine, University of Adelaide, Waite Campus, Glen Osmond, South Australia, Australia; 2 The James Hutton Institute, Invergowrie, Dundee, Scotland; 3 ARC Centre of Excellence in Plant Cell Walls, Centre for Nutrition and Food Sciences, Queensland Alliance for Agriculture and Food Innovation, University of Queensland, St Lucia, Queensland, Australia; 4 Division of Plant Sciences, School of Life Sciences, University of Dundee, Invergowrie, Dundee, Scotland; Institute of Genetics and Developmental Biology Chinese Academy of Sciences, CHINA

## Abstract

In barley endosperm arabinoxylan (AX) is the second most abundant cell wall polysaccharide and in wheat it is the most abundant polysaccharide in the starchy endosperm walls of the grain. AX is one of the main contributors to grain dietary fibre content providing several health benefits including cholesterol and glucose lowering effects, and antioxidant activities. Due to its complex structural features, AX might also affect the downstream applications of barley grain in malting and brewing. Using a high pressure liquid chromatography (HPLC) method we quantified AX amounts in mature grain in 128 spring 2-row barley accessions. Amounts ranged from ~ 5.2 μg/g to ~ 9 μg/g. We used this data for a Genome Wide Association Study (GWAS) that revealed three significant quantitative trait loci (QTL) associated with grain AX levels which passed a false discovery threshold (FDR) and are located on two of the seven barley chromosomes. Regions underlying the QTLs were scanned for genes likely to be involved in AX biosynthesis or turnover, and strong candidates, including glycosyltransferases from the GT43 and GT61 families and glycoside hydrolases from the GH10 family, were identified. Phylogenetic trees of selected gene families were built based on protein translations and were used to examine the relationship of the barley candidate genes to those in other species. Our data reaffirms the roles of existing genes thought to contribute to AX content, and identifies novel QTL (and candidate genes associated with them) potentially influencing the AX content of barley grain. One potential outcome of this work is the deployment of highly associated single nucleotide polymorphisms markers in breeding programs to guide the modification of AX abundance in barley grain.

## Introduction

In cereals, the arabinoxylan (AX) backbone consists of (1→ 4)-*β*-linked xylopyranosyl residues [1]. Glucuronic acid residues (sometimes 4-O-methylated) can be attached to the O-2 position on these backbone residues and α-L-arabinofuranosyl moieties are mainly attached to the O-3 position, making glucuronoarabinoxylans (GAX) [2]. GAX from the cell walls of barley aleurone and barley malt is highly substituted and carries arabinofuranosyl residues which can be attached at O-2, doubly linked to O-2 and O-3 or, as found most commonly, singly on the O-3 position [3,4]. While glucuronic acid and 4-O-methylated side chains are reported to be missing from the AX found in barley flour [5], barley husk contains AX with both 4-O-methylated glucuronic acid side chains at the O-2 position as well as arabinofuranosyl units linked to O-3 on the xylan backbone [6]. Acetyl subunits attached to O-2 and/or O-3 of the xylan backbone have been described in AX from wheat straw [7]. The presence of galactose and glucuronic acid substitutions on the AX extracted from Brewers' spent grain has been confirmed by methylation analysis [8] and suggests a more complex structure for barley grain AX than previously thought. Esterification of ferulic acid (FA) to the arabinofuranosyl side chains is considered to be a unique feature of cereal cell walls [9,10]. Cell walls of the aleurone from barley and wheat grain contain high levels of feruloylated AX causing a blue autofluorescence that can be easily detected under the microscope [11].

Cereals are the most widely cultivated crops globally, and the composition and structure of their cell walls have a significant effect on the end use of the grain. Plant cell walls are a major source of dietary fibre and antioxidants, they provide positive effects in human health and nutrition [12,13] and their structure and composition impact the use of grain in brewing, baking, in processed foods and for animal feed [14,15]. AX is the dominant non-cellulosic polysaccharide in the thick aleurone cell walls in barley grain [5], and is the second-most abundant component in the starchy endosperm cell walls after (1,3;1,4)-β-glucan [6]. (1,3;1,4)-β-Glucan constitutes around 75% of the barley starchy endosperm cell walls whilst AX contributes the majority of the remaining 25% of the cell wall matrix [7], while in wheat the converse is true [16]. The effects of higher or lower AX on downstream uses has attracted much less attention than the other major non-cellulosic polysaccharide (1,3;1,4)-β-glucan. However, evidence exists that AX might limit the extractability of (1,3;1,4)-β-glucan from barley grain [17]. This could be due to the fact that ferulic acid residues attached to the arabinosyl side chains can connect AX polysaccharides to each other, and potentially to other polymers through the formation of insoluble dehydrodimers [10,18]. There is evidence for interactions between alkali-extractable AX and (1,3;1,4)-β-glucan [19] whilst the loss of glucuronyltransferase activity in *Arabidopsis* gux1 and gux2 mutants which led to the absence of glucuronic or methylated glucuronic acid actually increased the extractability of xylan from cell walls [20]. Such reports support the hypothesis that the AX network can significantly influence the inter-molecular interactions and ultimate release of cell wall polysaccharides in cereal grain. This could be relevant to the germination process essential for seedling vigour and plant growth, to industrial processes such as malting, brewing and baking and to events in the human digestive tract where the availability of polysaccharides for microbial fermentation to short chain fatty acids is a key health determinant [21]. Additionally, AX is a major component of grain dietary fibre in cereals such as wheat and barley, and has the potential to provide health benefits which could reduce the chance of developing chronic conditions such as cardiovascular disease, diabetes and colon cancer [12, 13, 14, 21]. Also, the bioactive compounds, ferulic and p-coumaric acids, which are found esterified to the AX polymer have potential antioxidant activities [12, 13].

The biosynthetic machinery required for the synthesis of AX is complex and although there has been significant progress recently in gene identification and characterisation the function of many genes linked to the pathway remain to be definitively established. Members of the glycosyltransferase (GT) family 43 (GT43) in *Arabidopsis* have been shown to be involved in biosynthesis of the xylan backbone [1,22]. The *Arabidopsis Irregular Xylem* (*IRX*) mutants 9 and 14 (*irx9* and *irx14*) are members of the GT43 family exhibiting a dwarf phenotype with a reduction in xylosyltransferase activity [22–24]. The *irx10* mutant, a member of the GT47 family in *Arabidopsis* also exhibited a reduction in xylan content and xylosyltransferase activity similar to that of *irx9* and *irx14* [25]. The homologous genes *IRX9-Like* (*IRX9-L*), *IRX14-L* and *IRX10-L* also seem to be involved in xylan biosynthesis [22,23,25] whilst members of the GT8 family are implicated in the addition of glucuronic acid and methylated glucuronic acid residues to the xylan backbone [26]; *Arabidopsis gux1* and *gux2* mutants showed loss of xylan glucuronyltransferase activity[20]. Members of the DUF579 gene family have been associated with the methylation of glucuronic acid units [27] and mutations in some DUF579 genes, known as *IRX15* and *IRX15-L* in *Arabidopsis*, resulted in a decrease in xylan content and an increase in the degree of methylation [28,29]. UDP-xylose epimerases are involved in the interconversion of UDP-xylose and UDP-arabinose [30,31] whilst genes from the UDP-arabinose mutase family (also known as GT75) have been shown to be capable of converting UDP-arabinopyranose to the UDP-arabinofuranose form required for the biosynthesis of arabinosyl side chains [32–34]. Transfer of the arabinosyl units is believed to be mediated by GT61 genes [35–37] which comprises a very large gene family and a clade of acetyltransferases from the BAHD superfamily have been suggested to be involved in the feruloylation of arabinosyl side chains [38,39]. Acetylation of the xylan backbone is likely to be mediated by proteins from the DUF231 family [40,41]. Glycoside hydrolases (GH) with β-xylanase and arabinofuranohydrolase activities could also potentially be involved in the modification or turnover of AX [42,43].

Xylan synthesizing complexes (XSC) containing a number of protein types have been identified in wheat [33] and *Populous* [44] and most recently it was shown that three proteins from *Asparagus officinalis*, IRX9, 10 and 14 are required in a Golgi-localised XSC for xylan xylosyltransferase activity [45]. It is highly likely that other as yet unrecognised proteins are associated with such complexes, as has been found for cellulose [46,47].

Given the large number of structural genes required for the biosynthesis and modification of AX, the regulatory network is also expected to be complex. The activity of several key transcription factors and regulatory genes associated with secondary cell wall development and xylan biosynthesis in *Arabidopsis* have been described in the literature [48–50] but there is less information available for cereals in general. There has been less effort to identify genes associated with AX content of barley grain than in wheat starchy endosperm. Given the hexaploid nature of the wheat genome, gene identification in barley, which is closely related to wheat but diploid, is likely to be more straightforward, particularly with the availability of the barley genome [51]. Here a collection of 2-row spring barley cultivars was used to perform a Genome Wide Association Study (GWAS) in order to identify genes significantly influencing AX biosynthesis in whole barley grain. Ten genomic regions were found to be significantly associated with the AX content of barley grain and candidate genes for this trait were identified in these regions.

## Results and discussion

### Arabinoxylan content of barley grain

**Fig 1:**
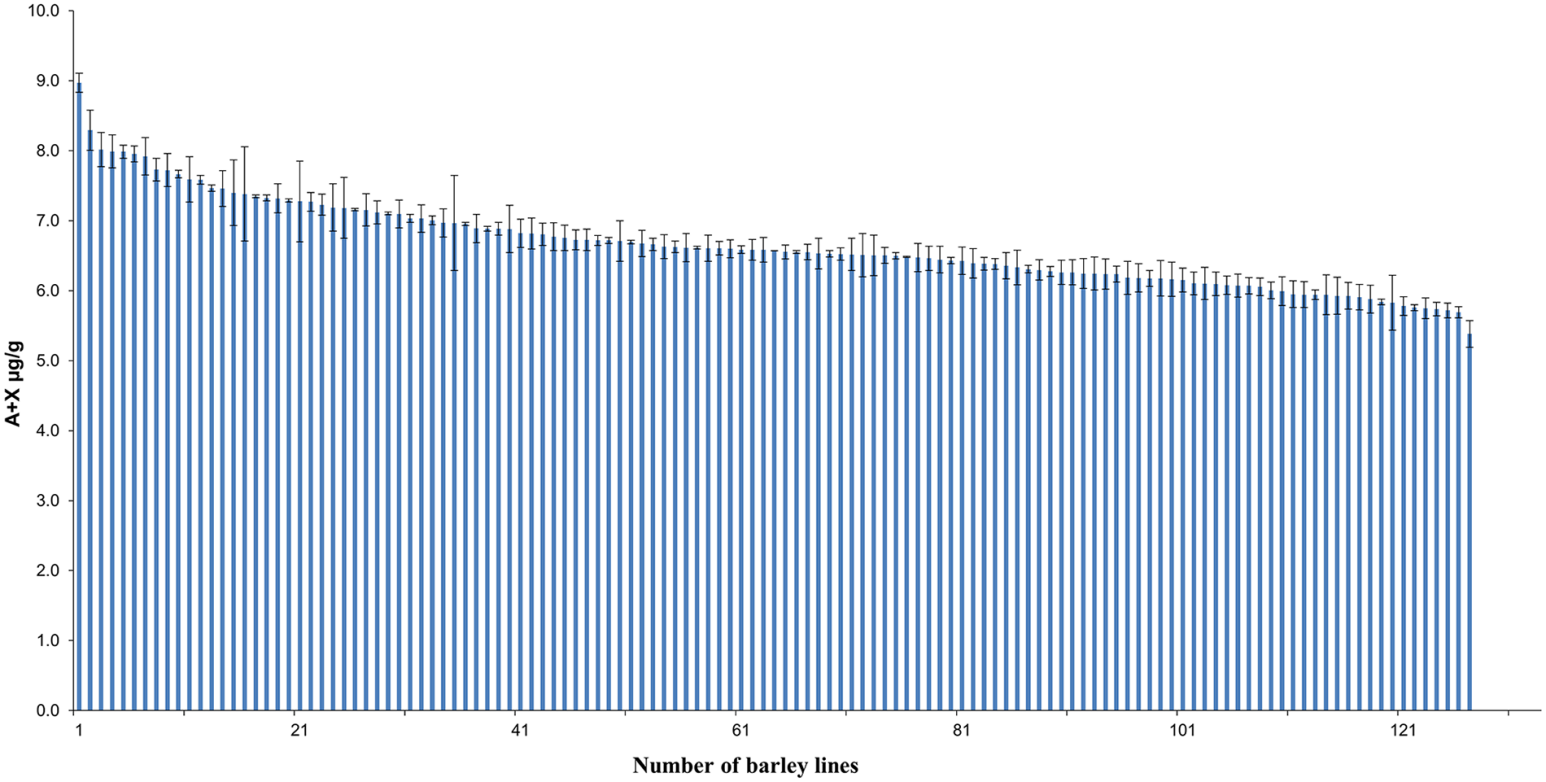
Arabinoxylan levels in wholegrain 2-row spring barley. Wholegrains of 128 glasshouse-grown lines were chemically analysed using monosaccharide analysis. Values represent the mean of the sum of arabinose (A) and xylose (X) expressed as w/w. Error bars represent standard deviation of the replicates.

The total grain arabinose plus xylose (A+X) content was quantified in 128 glasshouse grown 2-row spring barley accessions using HPLC analysis across two technical replicates. The large population size used in this study gave us the opportunity to explore natural variation in AX content of 2-row spring barley. An appreciable variation in AX content was observed in the barley grain where AX values expressed as weight/weight (w/w) ranged from 5.3 to ~ 9.0 μg/g (Fig 1) at an average value of 6.7 μg/g. Although these values are similar to those previously reported in the literature for barley grain (4.2–5.4% of dry weight) [52], the current study describes a wider range in barley grain AX content. Grains of other cereals exhibit such a dynamic range, including oat (4.1–14.5% of dry weight) and rye grains (8.0–12.1% of dry weight) [53,54], whilst wheat grain is reported to have an AX content of 5.5–7.8 (% of dry weight) [55]. A study on spring and winter wheat varieties also reported similar values for wheat grain AX content (4.4–6.9% of dry weight) [56]. In the current experiment we observed a higher grain AX content in our collection of barley accessions than that previously recorded for wheat. One reason for this comparably higher level of AX in barley could be the presence of the husk comprising the outer layers of the barley grain. Using four different chemical and enzymatic methods to study the monosaccharide composition of barley husk, it was shown that AX content of isolated fractions ranged from 50–83% [57]. Similar studies also showed that AX is the major polysaccharide found in barley husk, contributing to 45% of the total husk polysaccharide content [58]. A list of the germplasm used with corresponding AX levels is provided in S1 File.

### GWAS analysis

**Fig 2:**
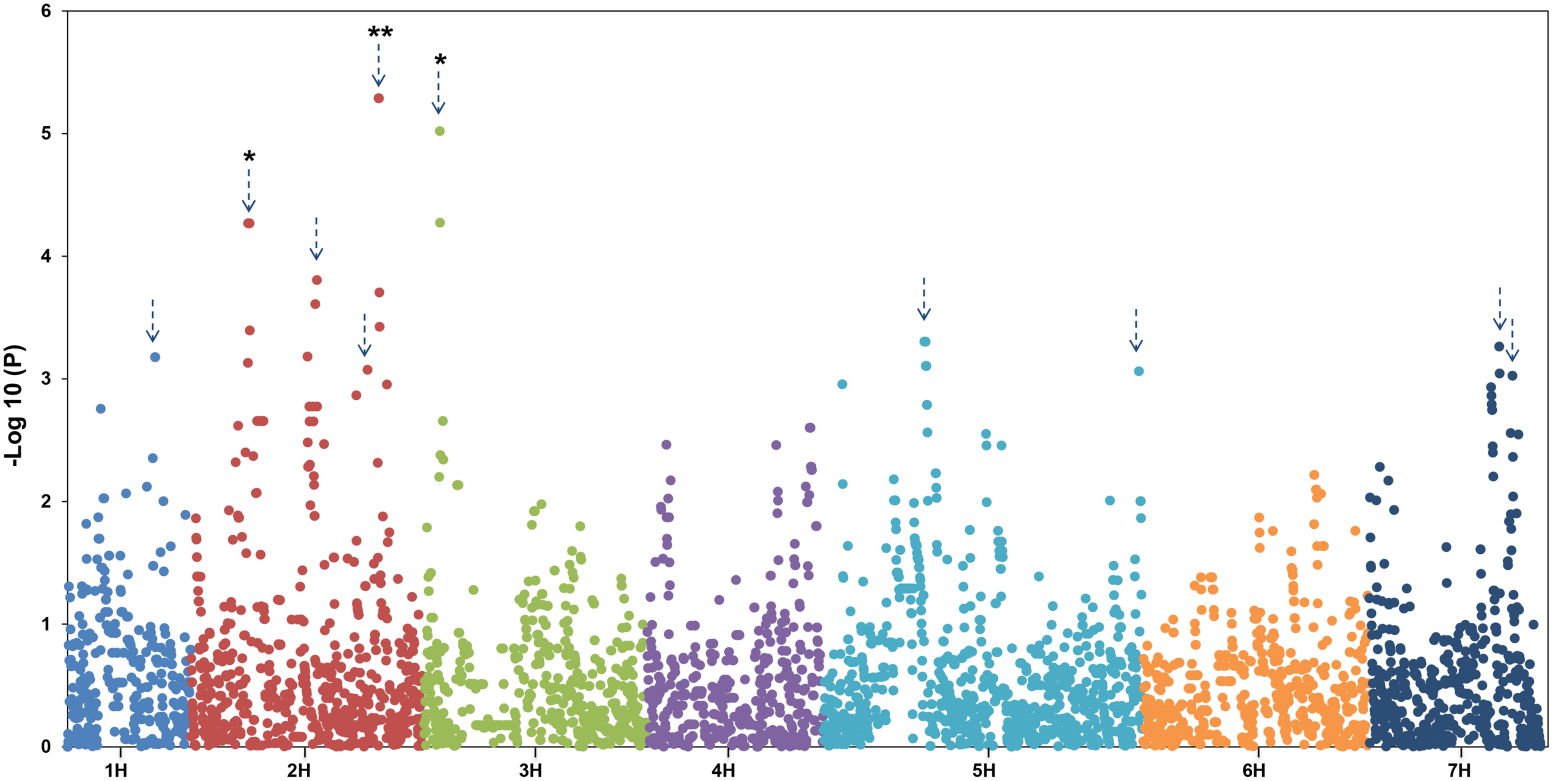
Manhattan plots of the GWAS of the wholegrain 2-row spring barley using the Eigenstrat model. The–Log 10 (P-value) is shown on the Y axis. The X axis shows the 7 barley chromosomes. Total AX content of wholegrain expressed as w/w was used for marker-trait association analysis.

Genome Wide Association Studies have become a common approach for gene identification in cereals. Recently, several studies successfully identified associations for cell wall polysaccharides including the (1,3;1,4)-β-glucan content of barley grain [59] and the AX content of tetraploid wheat [60]. In this study, we performed a GWAS on 2-row spring barley in an attempt to find regions significantly associated with the AX content of the grain. A total of 5182 SNP markers with a minimum allele frequency of >5% and less than 5% missing data were used to conduct the GWAS. We used an Eigenstrat model to account for population structure and to reduce the risk of false positive associations. Ten genomic regions were found to be significantly associated with barley grain AX content (Fig 2).

Three of the ten regions with a–Log10 (P)>3 (QAX2.S-2H1* (P> 0.05), QAX2.S-2H4** (P>0.01), and QAX2.S-3H1* (P> 0.05)) passed the more stringent FDR significance level (Table 1, Fig 2). The strongest QTL, QAX2.S-2H4**, is located on chromosome 2H (121–125 cM) with a–Log10–(P) value of 5.3 and an adjusted p value (q value) of 0.009. To search for genes within all intervals, we extended the intervals by 2.5 cM either side of the SNP with the highest LOD score. QAX2.S-2H1* and QAX2.S-2H4** contain members of gene families such as GT47 and GT61 (Table 1), for which there is now strong evidence for their role in contributing to AX content [25, 35]. For the 3 QTLS which passed the FDR we assessed the effect of the most significant SNP on grain AX content in the same collection of lines used to carry out the GWAS (S1 Fig). At QAX2.S-2H1, the average grain AX content varied by 0.86 μg/g depending on the allele of SCRI_RS_175065 present (t (4.28), p = 0.0085), where the average grain AX of accessions containing the adenine at this SNP was 6.59 μg/g compared to 7.45 μg/g in the accessions containing the alternate allele, a guanine.

**Table 1:** Significant associations correlated with barley grain AX content which passed the FDR test. Number of asterisks indicates the significance level for the adjusted P value (q value). Barley Gene id/ transcript (MLOC), Morex Contig, Developing grain without bracts 5 days post anthesis (CAR 5 DPA FPKM) and CAR 15 DPA FPKM (fragments per kilobase of exon per million fragments mapped) from ics.hutton.ac.uk/morexGenes.

chr	Peak	QTL position cM	Marker	i_select 9K (cM)	IBSC 2012 (cM)	MxBk POPSEQ 2013 (cM)	LOD	Gene	MxBk POPSEQ 2013 (cM)	contig	CAZY	PFAM	CAR 5	CAR 15
2H	QAX2.S-2H1*	50–55	SCRI_RS_175065	60.7	52.9	52.5	4.3	MLOC_17443	N/A	contig_1576718	GT-61	PF04577	1.6	1.0
								MLOC_77094	N/A	contig_74292	HvUAM2	PF03214	68.5	556.9
2H	QAX2.S-2H4**	121–125	SCRI_RS_221939	136.0	123.7	128.3	5.3	MLOC_72459	126.7	contig_61690	GT-43	PF03360	14.6	8.9
								MLOC_7681	125.3	contig_139847	GH-10	PF00331	0.0	0.1
								MLOC_4660	125.8	contig_135374	DUF579	PF04669	3.9	1.9
3H	QAX2.S-3H1*	13–19	SCRI_RS_192352	22.2	17.9	17.5	4.4	MLOC_75090	15.3	contig_67111	GH-10	PF00331	0.4	0.0

* > 0.05,

**> 0.01.

The allele of SCRI_RS_221939, which defines QAX2.S-2H4**, influenced average grain AX content by 0.70 μg/g (t (3.26), p = 0.0006) where accessions containing a cytosine had on average 7.27 μg/g grain AX, compared to those with a thymine at 6.57 μg/g. At QAX2.S-3H1, the average grain AX content varied by 0.38 μg/g depending on the allele of SCRI_RS_192352 present (t (3.70), p = 0.0032). At this SNP the accessions containing a cytosine had a higher average AX grain content (6.80 μg/g) compared to those containing a guanine (6.42 μg/g).

The output from the Eigenstrat analysis revealed that for SNP SCRI_RS_175065 (which represents QAX2.S-2H1) the lines containing the minor allele, at an allele frequency of 0.079, contributed to an increase in grain AX of 0.403 μg/g. For SNP SCRI_RS_192352, which represents QAX2.S-3H1, possession of the minor allele, which had a frequency of 0.378, was accompanied by a decrease in AX levels (-0.158 μg/g). Finally, for SNP SCRI_RS_221939, which represents the major QTL (QAX2.S-2H4**) on 2H, accessions that had the minor allele, with a frequency of 0.106, had a 0.358 μg/g higher grain AX level. The difference in effects for each of the SNP markers on AX levels, as indicated either by Eigenstrat analysis or the mean of differences provided with the boxplots in S1 Fig, are likely to be due to the corrections on population structure intrinsic to the Eigenstrat analysis.

The full list of QTL for grain AX content is provided in S1 Table, and will be described in the following sections. Very few of the genes identified in this study matched those identified in similar mapping experiments carried out on various wheat populations [60–62], making a comparison of candidate genes derived from this type of analyis unworkable at this stage.

### Genes associated with AX in barley grain

We identified candidate genes with known map positions corresponding to the genomic regions delineated by our association analysis. These included glycosyltransferase (GTs) and glycoside hydrolase genes (GHs) previously reported to be linked to AX biosynthesis and modification or hydrolysis. Also, genes involved in the biosynthesis of nucleotide sugar donors such as arabinose mutases and two families of genes with domains of unknown function, namely DUF231 and DUF579 were coincident with the QTL. A list of selected candidate genes identified under each significant association is provided in S1 Table. For all associations identified, 2.5 cM either side of the most significant marker were searched for likely candidate genes.

## Significantly associated biosynthetic genes

### Interconverting enzymes

Two genes from the UDP-arabinose mutase gene family (MLOC_77094 and MLOC_63185) were identified under QAX2.S-2H1 and QAX2.S-2H3 QTL respectively. These enzymes are central to the key conversion of UDP-arabinopyranose to UDP-arabinofuranose [34]. Notably these two genes have the highest transcript levels among all genes identified here at two stages of barley grain development (caryopsis 5 and 15 days post anthesis) (S1 Table).

### GT43

**Fig 3:**
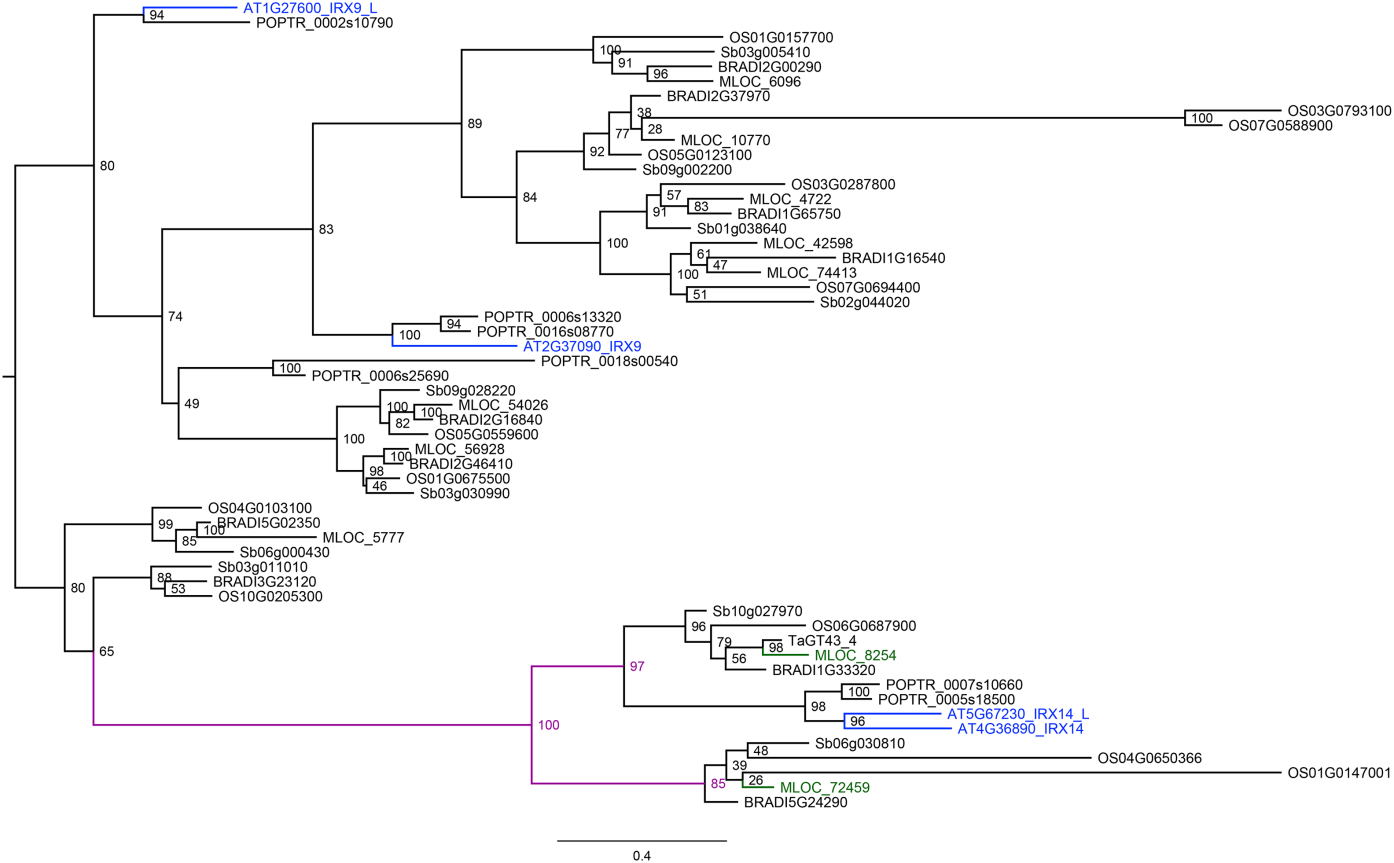
Phylogenetic tree of GT-43 genes from 6 species. Phylogenetic tree of GT-43 from *Arabidopsis* (At), barley (Hv), brachypodium (BRADI), sorghum (Sb), rice (OS) and the wheat TaGT43-4. Coding sequences of all genes were aligned using translation alignment. Phylogenetic trees were constructed using the FastTree function available in the Geneious software package. Branch labels represent FastTree support values. MLOC_ 8254 is on the same branch as the wheat TaGT43-4 and the *Arabidopsis* IRX14 and IRX14-L. MLOC_72459 is the only other barley gene closely related to the *Arabidopsis* IRX14 and IRX14-L. *Arabidopsis* IRX9 and IRX9-L are also shown in different colours.

Glycosyltransferase enzymes from a number of different families have been demonstrated to be central to xylan biosynthesis. Two members of the GT43 gene family, called *IRX9* and *IRX14* have been shown genetically to be non-redundantly involved in the elongation of the xylan backbone [63,64] but just a single GT43 gene was found under any of the ten associations identified here (S1 Table). Using a PFAM domain search (PF03360) we identified 10 GT43 proteins in barley, 13 in rice, 11 in sorghum, ten in *Brachypodium* and just four in *Arabidopsis*. Based on data from early to mid-caryopsis development available on the morexGenes- barley RNA-seq database (https://ics.hutton.ac.uk/morexGenes/index.html) we know that five of the ten GT43 genes in barley are expressed in one or both of these stages (S2 File). To establish how closely related the HvGT43 protein (MLOC_72459) under QAX2.S-2H4 is to the *Arabidopsis IRX9* and *IRX14*, or the related *IRX9-L* and *IRX14*-L genes, we produced a phylogenetic tree (Fig 3). This analysis was based on protein sequence translated from coding sequences of GT43 genes from various cereal species, including wheat GT43-4 that has been shown to be involved in biosynthesis of the GAX polymer [33]. It is clear that *At*IRX14 and *At*IRX14-L are closely related since they sit on neighbouring branches of a sub-clade which also contains wheat GT43-4, but not barley MLOC_72459 (Fig 3). Instead, a different barley protein from the GT43 family, located on chromosome 7H and not associated with any significant QTL, MLOC_8254, is most closely related to wheat GT43-4 and *Arabidopsis IRX14* and *IRX14*-L. Barley MLOC_72459 under QAX2.S-2H4 sits within a separate sub-clade (Fig 3). At the nucleotide level MLOC_72459 and MLOC_8254 share only 50.6% sequence identity (data not shown). The tree also indicates that *At*IRX9 and *A*tIRX9L are not closely related, fall into separate clades and neither closely match a cereal GT43. The lack of obvious orthologues of Arabidopsis IRX9 or IRX9-L may not be surprising when the structure of the xylans in cereals versus eudicots is considered, although this is likely to be more strongly linked to the nature of the substituents rather than intrinsic differences in the backbone [65,66]. Xylans are also present in more restricted tissues of eudicots and in smaller amounts than in cereals, however until the function of individual proteins is ascertained the reason for the presence or absence of particular orthologues is impossible to define. The importance of subtle differences could be key, for example expression of four rice GT-43 genes in Arabidopsis *irx9* mutants showed that only two genes (Os05g03174 and Os05g48600) were capable of restoring a wild type phenotype whilst one gene (Os06g47340) was capable of complementing the mutant phenotype of *irx14* [64].

### GT47

GT47 genes were found under two of the ten peaks (Table 1), one on 2H and one on 5H (MLOC_61178, and MLOC_12869). Genes in this family have been identified as *IRX10* or *IRX10-L*, and are xylan xylosyltransferases [67]. Downregulation of the Arabidopsis *IRX10* orthologue in rice resulted in a 10% decrease in xylan levels in stem cell walls [68]. RNAi silencing of *TaGT47-2*, the orthologue of IRX10 in wheat caused a dramatic decrease in AX content in transgenic lines as well as an increase in arabinosyl substitutions [69]. None of the GT47 genes in this study were identified as orthologues of *Arabidopsis IRX10* (data not shown) but nevertheless they remain candidates for AX biosynthesis in barley grain. Using a PFAM domain search for GT47 genes in barley (PF03016) we identified 30 family members, 10 of which are expressed in barley grain (S2 File). Given this large number of genes and the fact that the focus has been only on orthologous of *IRX10* in grasses [68,69], we were unable to draw any conclusions regarding the involvement of the GT47 genes identified here in the biosynthesis of AX polysaccharides in barley grain. A different approach such as RNA-Seq and transcript analysis by QPCR in an AX-depositing grass tissue could provide clearer evidence supporting the involvement of certain GT47 members in AX biosynthesis in grasses.

### GT61

Members of the GT61 family are being progressively identified as the proteins responsible for the addition of a range of xylan backbone substitutions in an increasing number of species [16,35–37,70]. Two genes from the GT61 gene family (MLOC_68728 and MLOC_17443) were identified in this study with MLOC_68728 located under QAX2.S-1H1 on 1H and MLOC_17443 found under QAX2.S-2H1*. Using a PFAM domain search tool we identified more than 30 members of the GT61 gene family in barley with at least 11 genes being expressed in developing barley grain (S2 File). Analysis of a rice mutant for a GT61 gene from the grass specific clade (xax1: Os02g22380) revealed that xylan from the mutant plants lacked β- Xylp-(1→2)-α-Araf-(1→3) structure substitutions, suggesting that Os02g22380 is a xylosyl transferase [36]. Interestingly, the mutant plants also lacked ferulic and p-coumaric acid, and exhibited an increase in the extractability of xylan and generally higher saccharification [36]. This was attributed to the lower degree of ferulic acid dehydrodimer cross-linking [36]. Further phylogenetic and transcript analysis of the GT61 genes identified here is required as these genes could be potential targets for modification of barley grain AX to enable increased release of xylan and other cell wall polysaccharides in a number of industrial processes.

### DUF579

**Fig 4:**
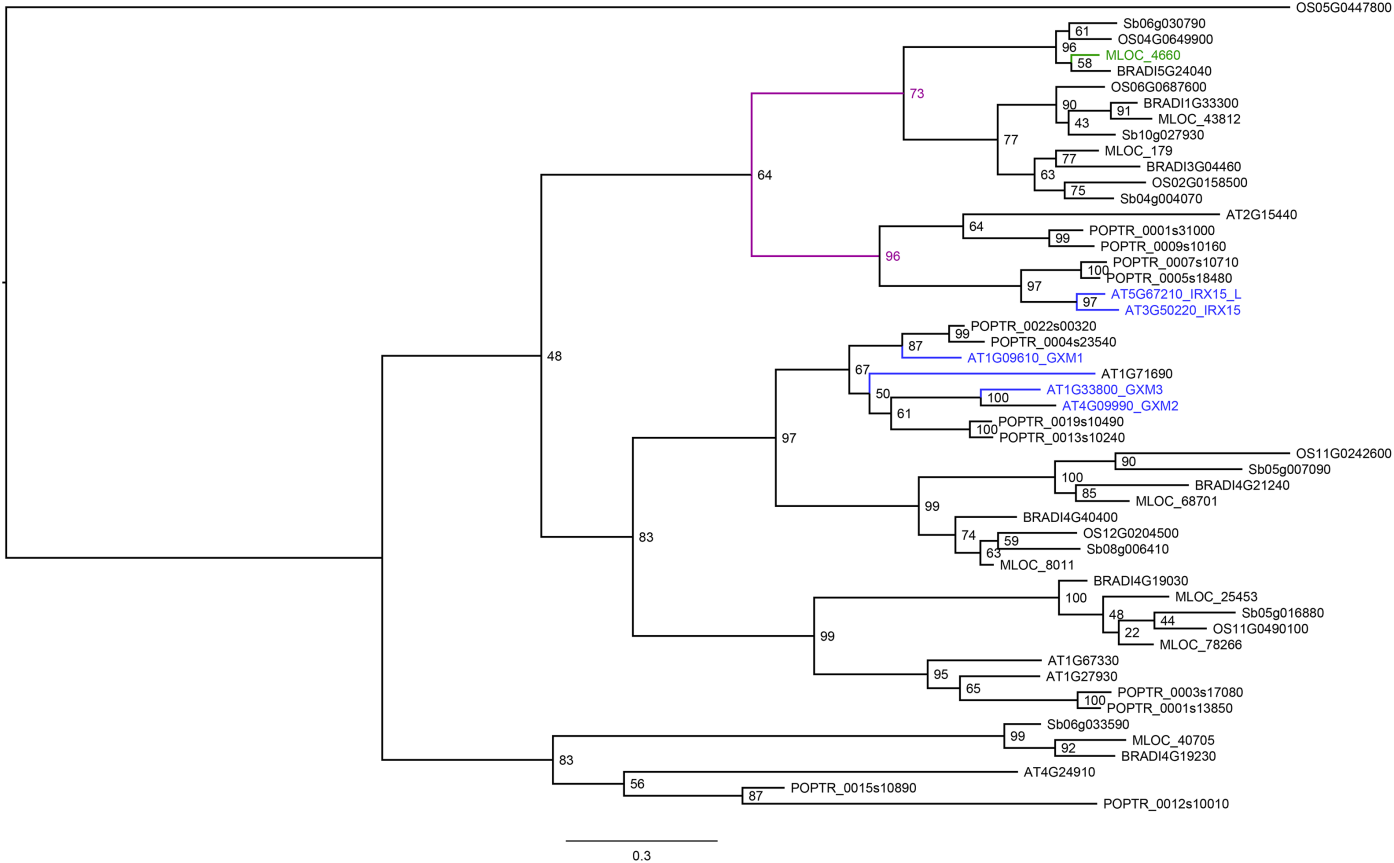
Phylogenetic tree of DUF579 genes from five species. Phylogenetic tree of DUF579 from *Arabidopsis* (At), barley (Hv), Brachypodium (BRADI), sorghum (Sb) and rice (Os). Coding sequences of all genes were converted using the translation alignment. Phylogenetic trees were constructed using the FastTree function available in the Geneious software package. Branch labels represent FastTree support values. *Arabidopsis* IRX15 and IRX15-L, and the barley gene MLOC_4660 identified in this study are shown in different colours.

A potential candidate for QAX2.S-2H4 is a DUF579 gene (MLOC_4660). Arabidopsis has ten members in this gene family, five of which are co-expressed with genes known to be involved in secondary cell wall development [29]. Two members of the DUF579 gene family in *Arabidopsis* known as *IRX15* (AT3G50220) and *IRX15-L* (AT5G67210) have been associated with xylan synthesis and deposition, as *irx15 irx15-L* double mutants exhibited irregular deposition of xylan in their secondary cell walls and contained xylan with a lower degree of polymerization [28,29]. However, three other members of this family, known as GXM1 (AT1G09610), GXM2 (AT4G09990) and GXM3 (AT1G33800), are believed to be involved in methylation of glucuronic acid residues attached to the xylan backbone [71]. Both a single mutation in AT1G33800 and double *gxm* mutants caused a significant reduction in xylan-bound methylated glucuronic acid [71]. Further characterization of AT1G33800 provided evidence that the protein encoded by this gene transfers a methyl group to α-D-glucopyranosyluronic acid residue linked to the xylan backbone [27]. Through a PFAM domain search (PF04669), eight DUF759 genes were identified in the barley genome, which were aligned with the four distinct phylogenetic clades that exist within the DUF579 gene family in *Arabidopsis* [27] and DUF579 proteins from poplar, rice, *Brachypodium* and sorghum. Our phylogenetic analysis also shows that DUF579 proteins fall into distinct clades (Fig 4). Certain members of the DUF579 proteins from all species included in the tree clustered with *Arabidopsis* IRX15 and IRX-15. Three barley proteins including MLOC_4660 clustered in a clade closely related to the *Arabidopsis* IRX15 and IRX15-L. However, it is not clear which barley gene is the orthologue of IRX15 or IRX15-L (Fig 4). Although experimental work has been carried out on DUF579 proteins from *Arabidopsis* and poplar [72,73], such information is still unavailable for members of this family from grasses. Whether MLOC_4660 is associated with xylan deposition, as it is the case with IRX 15 and IRX-15, is involved in methylation of glucuronic acid or plays a different role in AX biosynthesis needs to be further investigated. Nevertheless, this gene remains a potential candidate for the QAX2.S-2H4 QTL.

## Significantly associated modifying and hydrolytic genes

### Glycosyl hydrolases

It has been observed that there may be a finely tuned balance between biosynthetic and hydrolytic enzyme activity in the overall synthesis of a number of plant polysaccharides [74,75], although such hydrolases may also be key players in the modification and breakdown of these polymers. Members of the GH10 and GH11 [76,77], GH16 [78], GH51 [79,80] and GH79 [81] gene families have previously been associated with AX turnover and representatives were found under QAX2.S-3H1 (GH10; MLOC_75090), QAX2.S-5H1 (GH51; MLOC_56099 and GH79; MLOC_15027), and QAX2.S-5H2 (GH16; MLOC_80451). GH10 enzymes are endo-β-1, 4-xylanases and are involved in the hydrolysis of glycoside linkages of the xylan backbone [82]. Unlike xylanases from family 11 (GH11), GH10 xylanases may also be active on low molecular mass cellulose substrates [83]. However, both GH10 and GH11 xylanases are active on xylobiose and xylotriose substrates [84]. GH16 enzymes are mainly associated with the hydrolysis of (1,3–1,4)-β-glucan polysaccharides [85–87]. Based on the similarities in 3D structure between certain subgroups of GH16 enzymes and GH11 xylanases, it has been suggested that particular subgroups of GH16s might be active on AX [78]. However, this is yet to be functionally confirmed. GH51 enzymes are arabinofuranohydrolases, involved in the removal of arabinosyl side chains from AX [43]. GH79 enzymes exhibit a β-D-glucuronic acid activity [88] and thus could be involved in modification of GAX. Combined with other enzymes such as α-amylases, cellulases and pectinases, xylanases have important industrial applications in animal feed, food and drink and bread making industries [89], and thus offer a target for the manipulation of AX structure.

### GT31 and DUF231

A GT31 gene (MLOC_70708) was identified at QAX2.S-7H1. GT31 genes have been shown to have galactosyltransferase activity, and play a role in the biosynthesis of arabinogalactan peptides [90,91]. An α-L-galactopyranosyl-(1→2)-β-D-xylopyranosyl-(1→2)-5-O-trans-feruloyl-L-arabinofuranose structure has previously been reported for AX from maize bran [92], and has more recently been associated with the AX from other cereal grains, including barley [93]. Therefore it is possible that GT31 genes are involved in the transfer of galactosyl units during the biosynthesis of AX in barley grain and this may have an influence on the overall amounts in grain tissues.

One gene from the DUF231 (MLOC_81823) family was identified at QAX2.S-5H1, and some members of this family have been linked with the acetylation of the xylan backbone [94]. A double knockout of the *TBL32* and *TBL33* genes in *Arabidopsis* resulted in a significant decrease in xylan acetyl content [95], but had no effect on overall cell wall composition. However, there are indications that these genes, with many others, are under the control of the secondary wall master transcriptional regulators SND1 and NST1, the perturbation of which may lead to broad-reaching pleiotropic effects on cell wall composition and integrity.

## Conclusions

This GWAS study defined 10 QTL for the AX content of mature barley grain allowing candidate genes potentially involved in the biosynthesis of this important polysaccharide in cereals to be identified. Phylogenetic analysis of gene families suggest that a significant number of these genes may not be direct orthologues of AX-associated sequences in dicot plants such as Arabidopsis, indicating a need for further study of prime candidates, including transcript abundance and functional analysis. This could allow the use of promising candidates in conventional breeding efforts to manipulate AX levels in barley and other cereals, an industrially relevant goal for which there are currently few markers available.

## Materials and methods

### Plant material and growth conditions

A population of 2-row spring type barley was used in this study [96], (S1 File). This population comprised of 128 elite lines grown in a glasshouse compartment in a mix of clay-loam and cocopeat (50:50 v/v) at daytime and night-time temperatures of 22°C and 15°C respectively in The Plant Accelerator, Adelaide, Australia. This set was in particular selected to contain minimum population structure while maintaining as much diversity as possible based on population structure analysis and sequence homology. Mature grains were harvested and stored until monosaccharide analysis. For each line, five whole grains were ground to a fine powder using a ball mill (Mixer Mill MM400; Retsch Haan Germany) and the flour stored under dry conditions until the HPLC analysis.

### Genotyping of SNP markers

All lines were genotyped using the 9K iSelect SNP genotyping platform described previously [96]. Prior to marker-trait association analysis, all monomorphic markers with an allele frequency of > 95% and markers with missing data > 5% were excluded from the analysis.

### Monosaccharide analysis

A ~ 20 mg amount of wholegrain ground barley was used per sample. Monosaccharide analysis was carried out essentially as described by Comino et al. [97] with some modifications. Samples were treated with 1 mL 1 M sulphuric acid at 100°C for 3 hours. A 20-fold dilution of the hydrolysates was carried out prior to derivatization with 1-phenyl-3-methyl-5-pyrazolone (PMP). As an internal standard, 20 μL 0.5 mM 2-deoxy glucose was added to each sample. Excessive PMP was removed by dibutyl ether. A Phenomenex Kinetex 2.6 μm C18 100 × 3 mm 100A column installed on an Agilent 1260LC was used to separate the monosaccharides on an RP-HPLC. The flow rate was set to 0.8 mL/min. Eluents were (A) 10% acetonitrile, 40 mM ammonium acetate, and (B) 70% acetonitrile. The start condition was 85% A and 15% B and the gradient was 8 to 16% (B) over 12 mins. Detection was carried out at 250 nm. Calibration curves of standards of xylose and arabinose were used to quantify the area under the peaks.

### GWAS analysis

Marker-trait association analysis was carried out in GenStat 15^th^ Edition using the Eigenanalysis relationship model with a naïve model for comparison of each analysis (S2 Fig). For phenotype values, the mean values of the barley wholegrain total arabinose + xylose (w/w) was used. The false discovery rate (FDR) < 5% was calculated using the q value package in R [98] version 3.1.1. Boxplots to show the effect of SNPs from the three major QTL that passed the FDR test were produced using R version 3.2.2. To identify genes within intervals associated with AX content, the Barleymap website (http://floresta.eead.csic.es/barleymap/) was used. The intervals were extended by 2.5 cM either side of the SNP (s) with the highest LOD score to account for marker order uncertainty. SNPs significantly associated with the trait of interest within 5 cM of each other were considered to be linked to the same QTL and the SNP with the highest LOD score was used to represent the QTL. To obtain more consistent map positions, we compared the position of markers on three maps described in Comadran et al. [96], IBGS Consortium [51], and Mascher et al. [99]. QTL nomenclature is as described by Szűcs et al. [100] and available at (http://wheat.pw.usda.gov/ggpages/maps/OWB/).

### Bioinformatics and gene identification

Different tools were employed to find annotation for unknown genes under the intervals. For genes under the associations that had Accession numbers, the nucleotide sequences were downloaded from the NCBI database https://www.ncbi.nlm.nih.gov/gquery/) and then Blasted to the barley genome MLOC loci (http://plants.ensembl.org/index.html). The annotation for these MLOCs was established with a combination of PFAM analysis and by orthology to the other well annotated cereal genomes, *Brachypodium distachyon*, *Sorghum bicolor* and rice (*Oryza sativa*) (http://plants.ensembl.org/biomart/martview). MLOC numbers were used to search the morexGenes- barley RNA-seq database (https://ics.hutton.ac.uk/morexGenes/index.html) to identify potential *Arabidopsis* and or rice orthologs and also to download the transcript profile of the candidate genes across eight developmental stages. Other tools used included PFAM domain search (http://pfam.xfam.org/). The CAZY database (http://www.cazy.org/) was used as a reference for the potential glycosyltransferases (GT) and glycoside hydrolases (GH).

### Phylogenetic analysis

Amino acid sequences of barley, rice, sorghum and *Arabidopsis* glycosyltransferases were obtained from Ensemble Plants database (http://plants.ensembl.org/index.html) using a PFAM domain search. For GT43 the conserved PF03360 domain was used [101] and the protein sequence of TaGT43-4 described in Zeng et al. (ADK56174) [33] was included in the phylogeny analysis along with *Arabidopsis IRX14* (AT5G67230) and *IRX14-L* (AT4G36890), IRAX9 (AT2G37090) and IRX9-L (AT1G27600). The MLOC_72459 sequence from the (https://ics.hutton.ac.uk/morexGenes/index.html) was used in a blastn search against the barley nucleotide sequences available on the NCBI database to obtain a full length gene sequence. The amino acid sequence of this gene was aligned with other sequences from rice, sorghum, *Arabidopsis*, barley and wheat. The MUSCLE alignment tool available in the Geneious software package version 8.1.3. [102] was used to align all sequences, and gaps were deleted from the alignment. A phylogenetic tree of the alignment was then produced using the RAxML [103] tool available in the same software package. Protein model was set to GAMMA GTR with 1000 bootstraps. For DUF579, the PFAM PF04669 was used to search for members of this family in selected species.

## Supporting information

S1 File A list of all germplasm included in this study with their corresponding AX content. (CSV)

S2 File Total number of GT 47, GT 43, GT 8, GT 61, UAM and DUF579 genes in the barley genome identified through PFAM domain search. PFMA domains representing these families are listed in Table 1. Barley Gene id/ transcript (MLOC), Morex Contig, chromosomal location and developing grain without bracts 5 days post anthesis (CAR 5 DPA FPKM) and CAR 15 DPA FPKM (fragments per kilobase of exon per million fragments mapped) from ics.hutton.ac.uk/morexGenes.
(XLSX)

S1 Table Associations correlated with barley grain AX content identified in this study which passed the FDR test. A description of QTL and potential genes identified under the peaks that passed the FDR test. Number of asterisks indicates the significance level for the adjusted P value (q value). * > 0.05, **> 0.01, ***> 0.001.
(DOCX)

S1 Fig Mean effect on grain arabinoxylan content (μg/g) of alleles at the three SNPs which have the highest significance for the three QTLs which pass the FDR threshold. **A**. SCRI_RS_175065 (QAX2.S-2H1), **B**. SCRI_RS_221939 (QAX2.S-2H4), and **C**. SCRI_RS_192352 (QAX2.S-3H1*). p <0.01 = **, p<0.001 = ***.
(TIF)

S2 Fig Manhattan plots of the GWAS of the wholegrain 2-row spring barley using the null model. The–Log 10 (P-value) is shown on the Y axis. The X axis shows the seven barley chromosomes. Total AX content of wholegrain expressed as w/w was used for marker-trait association analysis.
(TIF)
