# Supplementary material for: A Genome Wide Association Study of arabinoxylan content in 2-row spring barley grain
Source: PLoS One. 2017 Aug 3;12(8):e0182537. doi: 10.1371/journal.pone.0182537 (PMC5542645; doi:10.1371/journal.pone.0182537)
Supplement: S1 Table — A description of QTL and potential genes identified under the peaks that passed the FDR test. Number of asterisks indicates the significance level for the adjusted P value (q value). * > 0.05, **> 0.01, ***> 0.001. (DOCX) [file pone.0182537.s003.docx]

| chr | Peak | QTL position  cM | Marker | i-select 9K | IBSC  2012 | MxBkPOPSEQ 2013 | LOD | Gene | MxBkPOPSEQ 2013 | contig | CAZY | PFAM | CAR  5 | CAR  15 |
| --- | --- | --- | --- | --- | --- | --- | --- | --- | --- | --- | --- | --- | --- | --- |
|  |  |  |  | (cM) | (cM) | (cM) |  |  | (cM) |  |  |  |  |  |
| 1H | QAX2.S-1H1 | 93- 98 | 11_20125 | 101.5 | 95.6 | 95.2 | 3.2 | MLOC_68728 | 100.5 | contig_55260 | GT-61 | PF04577 | 26.1 | 25.6 |
| 2H | QAX2.S-2H1* | 50- 55 | SCRI_RS_175065 | 60.7 | 52.9 | 52.5 | 4.3 | MLOC_17443 | N/A | contig_1576718 | GT-61 | PF04577 | 1.6 | 1.0 |
|  |  |  |  |  |  |  |  | MLOC_61178 | 57.1 | contig_45194 | GT-47 | PF03016 | 16.8 | 22.9 |
|  |  |  |  |  |  |  |  | MLOC_77094 | N/A | contig_74292 | HvUAM2 | PF03214 | 68.5 | 556.9 |
| 2H | QAX2.S-2H2 | 92-96 | SCRI_RS_135248 | 103.0 | 94.9 | 94.6 | 3.8 |  |  |  |  |  |  |  |
| 2H | QAX2.S-2H3 | 116-121 | 11_10109 | 126.5 | 118.7 | 114.0 | 3.1 | MLOC_63185 | 120 | contig_47354 | HvUAM4 | PF03214 | 141.1 | 129.3 |
| 2H | QAX2.S-2H4** | 121-125 | SCRI_RS_221939 | 136.0 | 123.7 | 128.3 | 5.3 | MLOC_72459 | 126.7 | contig_61690 | GT-43 | PF03360 | 14.6 | 8.9 |
|  |  |  |  |  |  |  |  | MLOC_7681 | 125.3 | contig_139847 | GH-10 | PF00331 | 0.0 | 0.1 |
|  |  |  |  |  |  |  |  | MLOC_4660 | 125.8 | contig_135374 | DUF579 | PF04669 | 3.9 | 1.9 |
| 3H | QAX2.S-3H1* | 13-19 | SCRI_RS_192352 | 22.2 | 17.9 | 17.5 | 4.4 | MLOC_75090 | 15.3 | contig_67111 | GH-10 | PF00331 | 0.4 | 0.0 |
| 5H | QAX2.S-5H1 | 43-50 | SCRI_RS_164250 | 50.0 | 46.5 | 47.0 | 3.3 | MLOC_12869 | 44.1 | contig_1564290 | GT-47 | PF03016 | 0.3 | 0.2 |
|  |  |  |  |  |  |  |  | MLOC_15026 | 44 | contig_1569647 | GT-8 | PF01501 | 23.2 | 15.2 |
|  |  |  |  |  |  |  |  | MLOC_15027 | 44 | contig_1569647 | GH-79 | PF03662 | 6.3 | 2.7 |
|  |  |  |  |  |  |  |  | MLOC_56099 | 44.1 | contig_40551 | GH-51 | PF06964 | 8.6 | 9.4 |
|  |  |  |  |  |  |  |  | MLOC_4908 | N/A | contig_135651 | GT-8 | PF01501 | 24.9 | 0.3 |
|  |  |  |  |  |  |  |  | MLOC_59956 | 47.6 | contig_44066 | GH-10 | PF00331 | N/A | N/A |
|  |  |  |  |  |  |  |  | MLOC_81823 | N/A | contig_98421 | DUF231 | PF03005 | 8.3 | 9.7 |
| 5H | QAX2.S-5H2 | 166-169 | SCRI_RS_4753 | 194.7 | 168.5 | 166.8 | 3.1 | MLOC_80451 | 167.7 | contig_9059 | GH-16 | PF00722 | 0.0 | 0.1 |
| 7H | QAX2.S-7H1 | 105-110 | 12_30164 | 120.3 | 108.1 | 106.4 | 3.3 | MLOC_70708 | 106.5 | contig_58412 | GT-31 | PF01762 | 14.4 | 10.5 |
| 7H | QAX2.S-7H2 | 118-123 | 11_10861 | 136.9 | N/A | 120.8 | 3.0 |  |  |  |  |  |  |  |

**Supplementary Table 1**. Significant associations correlated with barley grain AX content identified in this study.

A description of QTL and potential genes identified under the peaks. Number of asterisks indicates the significance level for the adjusted P value (q value). * > 0.05, **> 0.01, ***> 0.001. *. Barley Gene id/ transcript (MLOC), Morex Contig, Developing grain without bracts 5 days post anthesis (CAR 5 DPA FPKM) and CAR 15 DPA FPKM (fragments per kilobase of exon per million fragments mapped) from ics.hutton.ac.uk/morexGenes.
