# Supplementary figures and images for: A Genome Wide Association Study of arabinoxylan content in 2-row spring barley grain
Source: PLoS One. 2017 Aug 3;12(8):e0182537. doi: 10.1371/journal.pone.0182537 (PMC5542645; doi:10.1371/journal.pone.0182537)

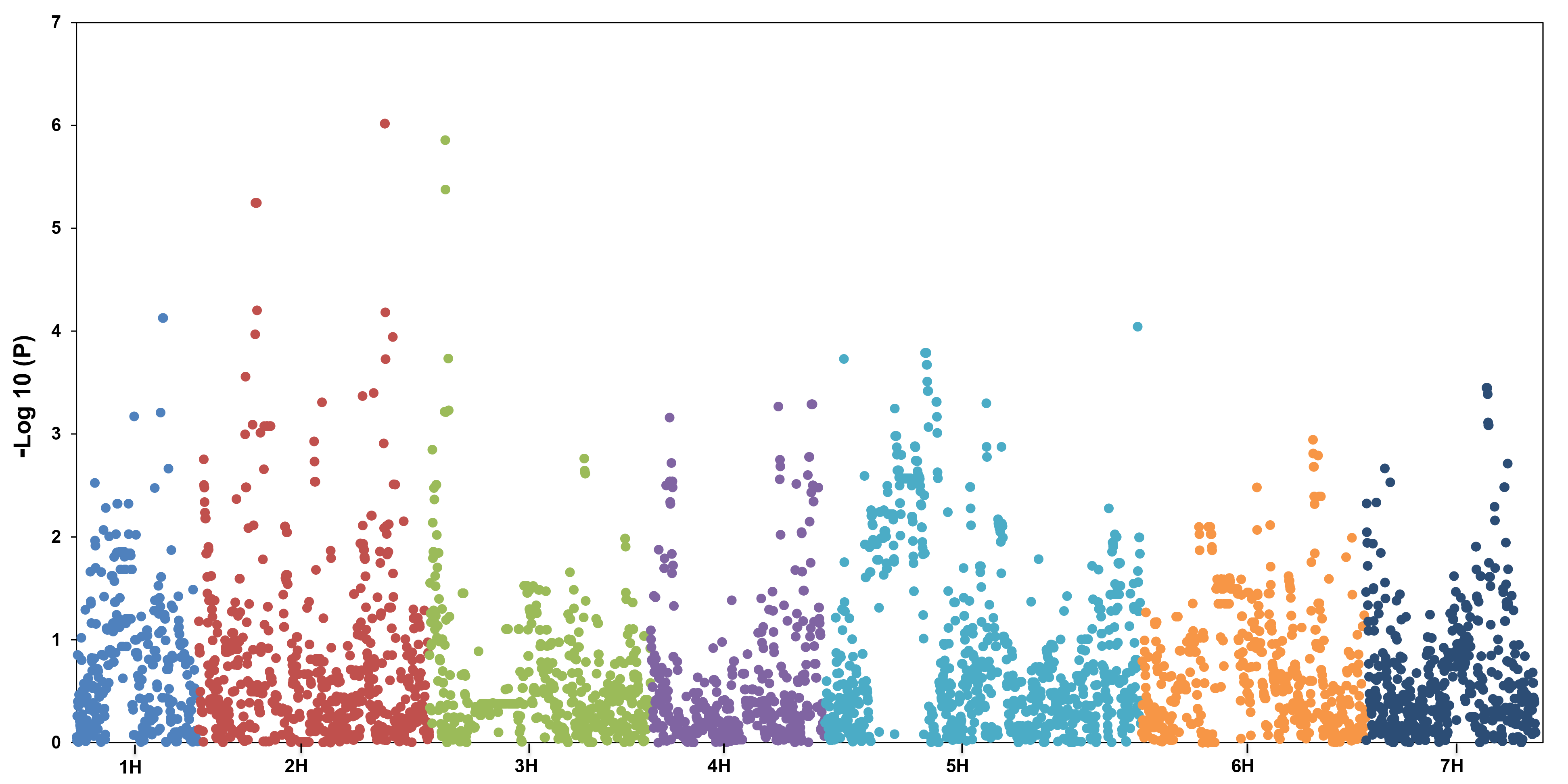

Supplement: S1 Fig — A. SCRI_RS_175065 (QAX2.S-2H1), B. SCRI_RS_221939 (QAX2.S-2H4), and C. SCRI_RS_192352 (QAX2.S-3H1*). p <0.01 = **, p<0.001 = ***. (TIF) [file pone.0182537.s004.tif]
